# Inflammation and the Peritoneal Membrane: Causes and Impact on Structure and Function during Peritoneal Dialysis

**DOI:** 10.1155/2012/912595

**Published:** 2012-03-25

**Authors:** Gilberto Baroni, Adriana Schuinski, Thyago P. de Moraes, Fernando Meyer, Roberto Pecoits-Filho

**Affiliations:** School of Medicine, Pontifical Catholic University of Paraná, State University of Ponta Grossa, 80215-901 Curitiba, PR, Brazil

## Abstract

Peritoneal dialysis therapy has increased in popularity since the end of the 1970s. This method provides a patient survival rate equivalent to hemodialysis and better preservation of residual renal function. However, technique failure by peritonitis, and ultrafiltration failure, which is a multifactorial complication that can affect up to 40% of patients after 3 years of therapy. Encapsulant peritoneal sclerosis is an extreme and potentially fatal manifestation. Causes of inflammation in peritoneal dialysis range from traditional factors to those related to chronic kidney disease *per se*, as well as from the peritoneal dialysis treatment, including the peritoneal dialysis catheter, dialysis solution, and infectious peritonitis. Peritoneal inflammation generated causes significant structural alterations including: thickening and cubic transformation of mesothelial cells, fibrin deposition, fibrous capsule formation, perivascular bleeding, and interstitial fibrosis. Structural alterations of the peritoneal membrane described above result in clinical and functional changes. One of these clinical manifestations is ultrafiltration failure and can occur in up to 30% of patients on PD after five years of treatment. An understanding of the mechanisms involved in peritoneal inflammation is fundamental to improve patient survival and provide a better quality of life.

## 1. Introduction

Peritoneal dialysis (PD) therapy has increased in popularity since the end of the 1970s. The method was developed as an alternative to hemodialysis (HD) presenting a patient survival rate equivalent to HD and better preservation of residual renal function. However, technique failure remains high, resulting in frequent modality changes. Currently, the two principal causes of technique failure in order of importance are (a) peritonitis, this important medical problem can also represent nearly 16% of the causes of death; (b) ultrafiltration failure, a multifactorial complication that can affect up to 40% of patients after 3 years of therapy [1].

**Figure 1:**
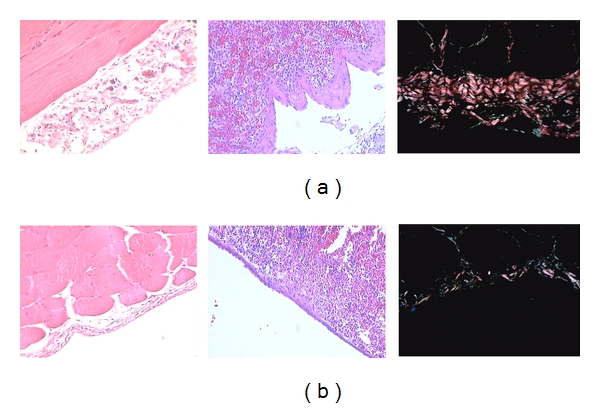
Typical alterations in the peritoneal membrane in an experimental model of hypertonic dialysate infusion (a) and the impact of oral statin use during 8 weeks of followup (b).

The peritoneal membrane is composed of different cell types with varying functions. Peritonitis as well as contact with bioincompatible solutions have deleterious effects on the membrane. These proinflammatory stimuli can induce lymphokine secretion by macrophages, which in turn, activate fibroblasts. Fibroblast activation has been associated with structural alterations in the peritoneal membrane of varying intensity. These alterations can be seen in Figure 1 which was extracted from a submitted study of our group. In this prospective controlled study in 20 nonuremic Wistar rats, peritoneal fibrosis occurs after exposure to glucose-based PD solutions and regardless the use of simvastatin.

Encapsulant peritoneal sclerosis (EPS) is an extreme and potentially fatal manifestation. EPS is a clinical syndrome that leads to persistent or recurrent intestinal obstruction, with or without inflammatory parameters of peritoneal thickening, sclerosis, calcification, and encapsulation, and can be inferred by clinical symptoms and radiology, but confirmed only by direct visualization with laparotomy [2, 3]. Incidence of EPS is heterogenous and has been reported to vary from 6 to 20% in eight years depending on the region.

## 2. Causes of Inflammation in PD

Causes of inflammation in peritoneal dialysis range from traditional factors to those related to chronic kidney disease *per se* as well as from the peritoneal dialysis treatment itself.

Uremia is a factor present in all PD patients and generates an inflammatory state causing stress on the peritoneum due to the formation of carbonyl products. It accelerates the formation of advanced glycation end products (AGEs) that induces an upregulation of the receptors of advanced glycation end products (RAGE) [4]. Dialysis decreases the impact of uremia, however, does not remove it completely.

The peritoneal dialysis catheter is the first proinflammatory factor associated to PD with which the patient comes into contact. After implantation in the peritoneum, the catheter can induce an inflammatory reaction as was demonstrated by Flessner et al. [5]. In addition, the catheter can occasionally be the site of bacterial biofilm formation.

 Initial therapy introduces the second inflammatory factor associated with PD: dialysis solution. Several PD solutions are available on the market today, and all are, to varying degrees, associated with peritoneal inflammation. Such inflammation is generated by several characteristics of these solutions, varying from low pH, presence of lactate, hyperosmolality, increased glucose concentration, presence of glucose degradation products (GDP) and advanced glycation end products (AGEs), and icodextrin metabolites, among others [6, 7].

Currently available glucose-based PD solutions present concentrations varying between 1.5 and 4.25% of glucose. The glucose load offered daily by a traditional PD prescription usually ranges from 120 g to 400 g.

The majority of PD solutions prescribed today markedly acidify pH to nearly 5.7 in approximately 2 to 3 minutes. This pH decreases viability of neutrophils and mesothelial cells, thus decreasing cytokine production and phagocytosis capacity. Lactate is utilized as a buffer in the majority of solutions. Its bioincompatibility with the peritoneal membrane is well known as well as its capacity to stimulate the production of fibroblast growth factors contributing to peritoneal fibrosis [8].

The association of icodextrin with EPS development is controversial. Some studies have associated the osmotic agent with EPS development [7], while others have shown it to be distinct, confirming its safety even with long-term utilization [6]. The relative rarity of the disease makes a definitive conclusion difficult. Even experimental studies with rats addressing this question are compromised by the increased *α*-amylase activity in these animals. The presence of this enzyme in plasma and in the peritoneal cavity provokes a rapid drop in peritoneal icodextrin concentration [9].

Chronic exposure to high glucose load in traditional PD solution induces significant inflammation of the peritoneal membrane. These solutions induce several proinflammatory factors such as PGA [10], vascular endothelial growth factors (VEGFs), fibroblast growth factor (TGF-*β*1), AGEs, and *upregulation* of RAGEs. Together, these factors contribute to the occurrence of neoangiogenesis and mesothelial fibrosis [11]. Glucose degradation products (GDPs), such as methylglyoxal, glyoxal, and 3-deoxyglucosone generated during the heat sterilization process, increase inflammation by inducing oxidative stress, which thus causes damage to mesothelial cells and leads to apoptosis and mesothelial denudation [12].

Substituting traditional solutions for more biocompatible solutions was recently associated with reduced membrane alterations [13]. It has been suggested for some years that the pathway of transforming growth factor *β*1/Smad plays a part in the development of peritoneal fibrosis. High glucose concentration in PD solutions is related to the activation of this pathway. The relationship between Smad2 and VEGF expression has also been reported. The latter is recognized as playing a role in angiogenesis, a histological characteristic that allows for differentiation from simple peritoneal fibrosis to EPS [14].

The endothelial system is another known factor with potent profibrotic characteristics and plays a role in the development of peritoneal fibrosis. This system can be activated by two receptors, endothelial receptors A and B. However, endothelial receptor B apparently does not play a role in peritoneal membrane thickening in experimental studies inducing deficiency of endothelial receptor B.

Finally, and of extreme importance, infectious peritonitis is an obvious cause of peritoneal inflammation and is associated with EPS development. Gram-positive organisms remain as the more prevalent peritonitis agents over the past decades representing up to 60% of cases followed by gram-negative organisms. However, the prevalence of peritonitis due gram-negative organisms is growing fast with the development of efficient strategies to control gram-positive infections. Despite all efforts made over the past decades, it still represents the most important cause of treatment discontinuation.

In sum, all the above-mentioned factors contribute to the release of proinflammatory cytokines such as interleukin 1*β* (IL 1*β*), tumor necrosis factor (TNF-*α*), IL-6, and IL-18. Structural lesions as a result of this process will be addressed below.

## 3. Structural Consequences of Inflammation of PD

Peritoneal inflammation generated by PD causes significant structural alterations in the peritoneum. These alterations, when severe, can trigger encapsulant peritoneal sclerosis [12]. Mesothelial exposure to PD solution in rats increased cytoplasm in these cells [15]. Thickening and cubic transformation of mesothelial cells occurs and is more accentuated in the parietal peritoneum [16]. Human peritoneal mesothelial cells (HPMCs) also suffer structural alterations and prominent transdifferentiation of HPMC to myofibroblasts occurs [17].

Histological alterations of the peritoneal membrane observed in EPS cases are nonspecific and are masked by the alterations commonly observed in patients with ultrafiltration failure and infectious peritonitis over the long term [18]. The most common findings are fibrin deposition, fibrous capsule formation, perivascular bleeding, interstitial fibrosis, and the presence of tissue granulation with vascular proliferation. Submesothelial tissue thickening also occurs with an increase in deposition of mesothelial conjunctive tissue [19, 20]. Fibrosis is characterized by the accumulation of extracellular matrix (ECM), resulting in disequilibrium between synthesis and degradation. Expression of collagen types 1 and 3 is significantly increased [21] as well as collagen type 4 [10]. Mesothelial cell denudation has also been described [22]. With respect to neoangiogenisis, we observed an arteriole diabetiform alteration and subendothelial hyalinosis of the venules [23]. 

## 4. Functional Consequence of Inflammation in PD

Structural alterations of the peritoneal membrane described above result in clinical and functional changes. One of these clinical manifestations is ultrafiltration (UF) failure and can occur in up to 30% of patients on PD after five years of treatment [1]. One of the presentations of UF failure occurs due to the increase in pores in the peritoneal membrane, which in turn accelerates small-solute transport dissipating the osmotic gradient necessary to maintain adequate fluid balance. This increase in vascular surface is observed in conjunction with an increase in density of interstitial fibers. These findings help justify the increase in transport of small molecules, while the alterations in the UF coefficient are only moderate [24]. In addition to UF failure, clinical manifestations such as severe malnutrition, subocclusion or intestinal occlusion, and ascites suggest the presence of EPS even after discontinuation of PD.

 Prescribing more hypertonic glucose solutions is a common strategy to counter this drop in UF, primarily where there is no available icodextrin. This intensifies and perpetuates inflammatory disturbances, with a direct impact on dialysis adequacy and fluid balance. The final consequence is the inevitable transfer to HD. Despite all damage to the peritoneal membrane with therapies performed today, large observational studies have shown an important evolution in PD patient survival when compared to HD over the past years [25].

## 5. Conclusion

PD initiation increases inflammatory stimuli for the chronic kidney patient such as the presence of the peritoneal catheter, use of bioincompatible solutions, and possible infectious peritonitis. Together, these factors generate structural and physiological alterations of the peritoneal membrane. These manifestations are frequently observed and can range from difficulties in obtaining an adequate fluid balance until the dreaded encapsulant peritoneal sclerosis. Nevertheless, patient survival in PD is similar to that of HD. An understanding of the mechanisms involved in peritoneal inflammation is fundamental for the development of new strategies. This knowledge can provide not only a better technique survival, but also improvements in patient survival and a better quality of life.
